# Role of bispectral index monitoring and burst suppression in prognostication following out-of-hospital cardiac arrest: a systematic review protocol

**DOI:** 10.1186/s13643-017-0584-6

**Published:** 2017-09-25

**Authors:** Leanne Eveson, Marcela Vizcaychipi, Shashank Patil

**Affiliations:** 1 Emergency Medicine, Chelsea and Westminster Hospital NHS Foundation Trust, 369 Fulham Road, London, SW10 9NH UK; 2 Anaesthesia & Intensive Care Medicine, Chelsea and Westminster Hospital NHS Foundation Trust, 369 Fulham Road, London, SW10 9NH UK

**Keywords:** Bispectral index, BIS, Burst suppression, Out-of-hospital cardiac arrest, OHCA, Prognostication

## Abstract

**Background:**

Out-of-hospital cardiac arrest (OHCA) is associated with significant mortality or may have a poor neurological outcome. Various community-training programmes have improved practices like bystander cardiopulmonary resuscitation (CPR) and early defibrillation using automated external defibrillator (AED). Post-resuscitation care has also changed significantly in the millennium. Interventions like targeted temperature management (TTM), avoidance of hyperoxia and emergency cardiac catheterisation have given patients a chance of a better neurological outcome. Despite these timely interventions, it is still very difficult to predict neurological outcome. The European Resuscitation Council and European Society of Intensive Care Medicine (ERC-ESICM) published guidance in 2015 with a strong recommendation to delay prognostication for at least 72 h and with an emphasis to adapt a multimodal approach, which includes neurological examination, biomarkers, electroencephalogram (EEG) and radiological tests. These interventions not only have cost attached to them, but the unpredictability has a significant emotional impact on family members. Bispectral index (BIS) monitoring device acts on the principle of EEG and converts the waveform into an absolute number and also measures the burst suppression. We hypothesize that patients who have a low BIS value and high burst suppression within 24 h of presentation will have a poor neurological outcome. The primary objective of this review is to look at BIS monitor as a tool, which could help bring forward the timing of prognostication.

**Methods:**

Electronic databases will be systematically searched for randomised controlled trials and prospective or retrospective cohort studies with no language restrictions. The search will be supplemented with grey literature searches of thesis, dissertations and hand searching of relevant journals. Two independent reviewers will screen, select and perform analysis according to the Preferred Reporting Items for Systematic Review and Meta-analysis (PRISMA) method. The selected studies will be analysed using the Grades of Recommendation, Assessment, Development and Evaluation (GRADE) system. Meta-analysis will be performed if suitable.

**Discussion:**

This review will synthesize the evidence on the use of BIS monitors within 24 h of achieving return of spontaneous circulation (ROSC) and may help in early prognostication.

**Systematic review registration:**

PROSPERO CRD 42016050224.

**Electronic supplementary material:**

The online version of this article (10.1186/s13643-017-0584-6) contains supplementary material, which is available to authorized users.

## Background

Out-of-hospital cardiac arrest (OHCA) is associated with a high mortality. Average survival to hospital discharge for OHCA patients in England in 2013 was only 8.6% [1]. Despite advances in post-resuscitation care management, about 50% of resuscitated patients from cardiac arrest (CA) die or have a poor neurological prognosis. One of the major causes of mortality following CA is severe neurological damage due to post-anoxic brain injury [2]. The cost and length of stay is higher in patients with poor neurological outcome, which can be up to £50,000 per survivor [3]. There are further considerations like community care and rehabilitation, quality of life and emotional impact on the family. It is therefore essential to predict neurological outcome in this group of patients as early as possible, in order to potentially enable early withdrawal of life-saving treatment (WLST) in those patients predicted to have a poor outcome.

Cardiac arrest causes a significant pathological and clinical impact, known as ‘post-cardiac arrest syndrome’ [4].

The four key components of post-cardiac arrest syndrome were identified as:

I. Post-cardiac arrest brain injury;
II. Post-cardiac arrest myocardial dysfunction;
III. Systemic ischaemic / reperfusion injury;
IV. Persisting precipitating pathology

Post-resuscitation care aims to reduce this impact; it has developed and evolved significantly since 2003, following recommendations by the Advanced Life Support (ALS) task force of the International Liaison Committee on Resuscitation (ILCOR) to implement Therapeutic Hypothermia (TH) in unconscious survivors following OHCA [5]. The 2015 European Resuscitation Council (ERC) and European Society of Intensive Care Medicine (ESICM) guidelines on post-resuscitation care made strong recommendations to avoid severe hyperoxia (large amounts of oxygen) for patients following CA [6–8], emergency cardiac catheterisation ± immediate percutaneous coronary intervention (PCI) and targeted temperature management (TTM) between 32–36 °C [9].

In 2015, ERC-ESICM recommended a multimodal prognostication approach for comatose survivors following CA [10]. It was based on a robust analysis of evidence and provided a practical recommendation. Hence, it formed the basis of ERC guidelines on resuscitation published in 2015.

The key recommendations are summarised below with a great emphasis on the fact that they should be used in conjunction with each other:

1. Clinical examination

- Using bilateral pupillary and corneal reflexes at 72 h or more from return of spontaneous circulation (ROSC) to predict poor outcome in comatose survivors from cardiac arrest, either TH or non-TH treated patients;

2. Myoclonus and status myoclonus

- Using the term status myoclonus to indicate a continuous and generalised myoclonus persisting > 30 min in comatose survivors of CA;
- Using the presence of a status myoclonus within 48 h from ROSC in combination with other predictors to predict poor outcome in comatose survivors of CA, either TH or non-TH treated.

3. Bilateral absence of SSEP (somatosensory-evoked potentials) N20 wave

- Using bilateral absence of SSEP N20 wave at ≥ 72 h from ROSC to predict outcome in comatose survivors following CA treated with controlled temperature;
- There was suggestion to use SSEP at ≥ 24 h from ROSC to predict outcome in comatose survivors following CA not treated with controlled temperature.

4. Electroencephalogram (EEG)

- Absence of EEG reactivity to external stimuli, presence of burst suppression or status epilepticus at ≥ 72 h after ROSC to predict poor outcome in comatose survivors from CA.

5. Biomarkers

- There is suggestion to use high NSE (neuron-specific enolase) at 48–72 h from ROSC in combination with other predictors for prognosticating a poor neurological outcome in comatose survivors following CA, either TH or non-TH treated.

6. Imaging

- Using the presence of a marked reduction in grey matter/white matter (GW/WM) ratio or sulcal effacement on brain CT within 24 h after ROSC or presence of the extensive reduction in diffusion on brain MRI at 2–5 days after ROSC to predict a poor outcome in comatose survivors following CA both TH or non-TH treated.

The quality of evidence on which the above strategy is developed and recommended is low to very low. Golan et al. in their meta-analysis showed that only three tests accurately predicted poor prognosis with low false positive rates (FPR): bilateral absences of pupillary reflex more than 24 h after CA (FPR 2%, confidence interval (CI) 1–6%), bilateral absence of corneal reflex more than 24 h post CA (FPR 4%, CI 1–9%) and bilateral absence of SSEP between day 1 and 7 (FPR 3%, CI 1–7%). FPR were higher for a Glasgow Coma Score-Motor response (GCS-M) less than 2, unfavourable EEG patterns, myoclonic status epilepticus and elevated NSE [11].

Also, despite the recommended delayed prognostication strategy, a large single-centre study of 326 patients found that 30% of patients have delayed awakening (i.e. still in coma after TTM and sedation withdrawal) and up to 20% remained comatose at 1 week [12].

There are other modalities like bispectral index (BIS) monitor and infrared pupillometry, which are currently being trialled to predict neurological outcome [13–15]. These are non-invasive techniques and can be used in emergency or intensive care settings with a certain degree of training. We suggest, given the available low quality of evidence and recommended multimodal approach, that there is definitely a place for a new modality. This study aims to look at the available evidence to support early use of Bispectral Index and burst suppression (BR) monitoring especially in the emergency department (ED) to help predict neurological outcome.

### BIS monitor

BIS monitor is the brain monitoring system for critical care developed by Covidien-Medtronic. A sensor is placed on the patient’s forehead and raw electroencephalogram (EEG) data is collected. The EEG information is processed by the system and calculates a number between 0 and 100. This provides a direct measure of a patient’s level of consciousness. See Table 1 for interpretation of BIS value and clinical state.

**Table 1:** BIS value and clinical states

BIS index range	Clinical state
100	Awake: responds to normal voice
80	Light to moderate sedation: may respond to loud commands
60	General anaesthesia
40	Deep hypnotic state
20	Burst suppression
0	Flat-line EEG

### BSR

During the burst suppression phase, suppression wave (amplitude < 0.5 μV) follows burst wave (amplitude > 0.5 μV). The suppression ratio is expressed as a percentage and is the ratio of total duration of suppression wave to total duration of analysis [16].

### Primary hypothesis

In a patient who remains comatose following ROSC after cardiac arrest, low BIS value (< 20) and high burst suppression ratio is a predictor of poor neurological outcome. The aim of this study is to analyse the available evidence to support early use of BIS and BSR monitoring in the ED to help predict neurological outcome.

## Methods

The systematic review will be designed in accordance with the Preferred Reporting Items for Systematic Reviews and Meta-analyses (PRISMA-P statement) [17]. The systematic review protocol is provided in Additional file 1. This protocol is registered with PROSPERO as CRD42016050224.

### Eligibility criteria

#### Study design

This systematic review will include randomised controlled studies and prospective and retrospective cohort studies. A grey literature search of thesis and dissertations and hand searching of relevant journals will also be carried out. The study will include adult (age greater than or equal to 18 years) cardiac arrest patients. All arrest rhythms will be included. Studies will be included if they report the use of BIS monitoring and/or BSR monitoring and if the BIS and BSR monitoring were initiated within 24 h of ROSC. Studies will be included in which the reported outcome of interest is neurologically-intact (cerebral performance category CPC 1–2). Studies will be excluded if the outcome of interest is survival rather than performance status, if BIS or BSR monitoring was commenced during resuscitation effort or commenced later than 24 h following ROSC, and all case reports will be excluded.

#### Information sources

Studies will be identified through a systematic search of the following electronic databases: MEDLINE (Ovid), EMBASE (Ovid), CINHAL (Ebsco), Cochrane Database of Systematic Reviews (CDSR), DARE (Database of Abstracts of Reviews of Effectiveness), LILACS (Latin American and Caribbean Health Sciences Literature), International Clinical Trials Registry Platform (ICTRP), Science citation index-expanded (SCI-expanded) and Conference Proceedings Citation Index-Science (CPCI-S), as well as clinical trials, dissertations and theses. PROSPERO repository will also be searched for all active or completed systematic reviews. Searches of electronic databases will be supplemented with discussions with authors of unpublished studies, inspection of reference lists of relevant articles and hand searching of pertinent journals. As BIS was introduced in 1994, searches from 1994 onwards are considered sufficient.

#### Search strategy

The search strategy was designed and conducted by an information specialist experienced in systematic reviews (PB) following the Cochrane handbook for systematic review of interventions [18]. The search includes controlled vocabulary (MeSH) and natural language terms: Bispectral index, BIS, burst suppression, out-of-hospital cardiac arrest, OHCA and prognostication. If a study is considered partially eligible based on the abstract, we will attempt to extract the required data. If this cannot be done, we will include this in the report of the review. The final search strategy will be peer-reviewed by an independent medical information specialist using the Peer Review of Electronic Search Strategies (PRESS) checklist [19]. No language restriction will be applied. The search will be updated at the end of our review to ensure the most recent relevant articles are included. A detailed search strategy for MEDLINE and EMBASE is provided at Additional file 2.

#### Data management

The results of the literature search will be stored in HDAS (Healthcare Databases Advanced Search) and screened according to the study selection process. The selected studies will be analysed using the Grades of Recommendation, Assessment, Development and Evaluation (GRADE) system [20]. The data extraction template for Cochrane reviews [21] will be used to create the study eligibility form, please see Additional file 3.

#### Study selection

A hierarchical screening method adapted from PRISMA will be used by two reviewers, SP and LE. The reviewers will independently screen the titles and abstracts highlighted by the search strategy. Eligible studies will be selected for full text analysis. Reviewers will record reasons for exclusion of studies in a data extraction form based on the data extraction template for Cochrane reviews. Authors will not be blinded to the journal titles, authors or institutions.

The following data will be extracted from each eligible study: patient age, survival to discharge, use of therapeutic hypothermia, use of neuromuscular blockade, BIS/BSR use, BIS level and/or BSR for those with CPC 1–2 versus CPC 3–5, time point of BIS monitoring, sensitivity and specificity of BIS level studies and primary outcome. Study design, sample size, year of study conduct, year of study publication and country of origin will also be recorded.

If required, authors of eligible studies will be contacted for discussion. All discussions with authors will be documented. Data extracted will be collected and organised in a Microsoft Excel spreadsheet.

### Risk of bias and quality assessment of individual studies

All eligible studies will be assessed for risk of bias (RoB) using the RoB in non-randomised studies of interventions (ROBINS-I) tool [22] and the Cochrane RoB tool for randomised controlled trials [23]. Disagreements between reviewers will be recorded and discussed and resolved by an independent reviewer.

### Statistical analysis

The aim of this review is to determine the association between patients who have a low BIS value and high BSR within 24 h of presentation post OHCA and neurological outcome. The results from all studies will be included in statistical analysis. Odds ratios will be used to examine the role of BIS as a prognostic factor for neurological outcome. We will calculate pooled estimates of effect of intervention, together with *p* values and confidence intervals. Variation will be checked for between studies (heterogeneity) and we will also analyse publication bias. We assume there will be heterogeneity due to limited evidence in this field and will apply the random-effects model [24]. The methods that will be used to test for heterogeneity are *I*-square statistics [25]. If significant heterogeneity is found, we will perform a sensitivity or sub-group analysis. Meta-regression will be used to examine the causes of any significant heterogeneity.

## Discussion

OHCA is associated with significant mortality or may have a poor neurological outcome. Prognostication of OHCA survivors poses a significant challenge. The current delayed multimodal prognostication model is recommended on the basis of currently available low to very low quality of evidence. BIS monitoring is currently not widely used; however, it is relatively simple to use, easy to train operators, non-invasive, can be used in emergency and intensive care settings and multiple readings can be available at various timelines during post-resuscitation care management. This review will synthesize the evidence available on the use of BIS monitors at various timelines after achieving ROSC and may help contribute as an additional modality to the current multimodal prognostication model.

## Additional files

Additional file 1: PRISMA-P (Preferred Reporting Items for Systematic review and Meta-Analysis Protocols) 2015 checklist: recommended items to address in a systematic review protocol. (PDF 145 kb)

Additional file 2: Search Strategy on MEDLINE and EMBASE. (PDF 97 kb)

Additional file 3: Cochrane public health group data extraction and assessment template (DOCX 93 kb)

## Abbreviations

AED: Automated external defibrillator
ALS: Advanced life support
BIS: Bispectral index
BSR: Burst suppression ratio
CDSR: Cochrane Database of Systematic Reviews
CPC: Cerebral performance category
CPCI-S: Conference Proceedings Citation Index-Science
CPR: Cardiopulmonary resuscitation
CT: Computed tomography
DARE: Database of Abstracts of Reviews of Effectiveness
ED: Emergency Department
EEG: Electroencephalogram
ERC: European Resuscitation Council
ERC-ESICM: European Resuscitation Council and European Society of Intensive Care Medicine
ESICM: European Society of Intensive Care Medicine
FPR: False positive rates
GCS-M: Glasgow Coma Score-Motor response
GM/WM: Grey matter/white matter
GRADE: Grades of Recommendation, Assessment, Development and Evaluation
HDAS: Healthcare Databases Advanced Search
ICTRP: International Clinical Trials Registry Platform
ILCOR: International Liaison Committee on Resuscitation
LE: Leanne Eveson
LILACS: Latin American and Caribbean Health Sciences Literature
MeSH: Medical subject headings
MRI: Magnetic resonance imaging
MV: Marcela Vizcaychipi
NSE: Neuron-specific enolase
OHCA: Out-of-hospital cardiac arrest
PCI: Percutaneous coronary intervention
PRESS: Peer Review of Electronic Search Strategies
PRISMA: Preferred Reporting Items for Systematic Review and Meta-analysis
RoB: Risk of bias
ROBINS-I: Risk of bias in non-randomised studies of interventions
ROSC: Return of spontaneous circulation
SCI: Science citation index
SP: Shashank Patil
SSEP: Somatosensory-evoked potentials
TH: Therapeutic hypothermia
TTM: Targeted temperature management
WLST: Withdrawal of life-saving treatment
