# Supplementary material for: Role of bispectral index monitoring and burst suppression in prognostication following out-of-hospital cardiac arrest: a systematic review protocol
Source: Syst Rev. 2017 Sep 25;6:191. doi: 10.1186/s13643-017-0584-6 (PMC5613623; doi:10.1186/s13643-017-0584-6)
Supplement: Supplementary file 2 — Search Strategy on MEDLINE and EMBASE. (PDF 97 kb) [file 13643_2017_584_MOESM2_ESM.pdf]

#	Database	Search term	Results
1	Medline	(bispectral index).ti,ab	2650
2	Medline	(BIS).ti,ab	80553
3	Medline	"MONITORING, INTRAOPERATIVE"/ OR "CONSCIOUSNESS MONITORS"/	17217
4	Medline	(bispectral index monitor).ti,ab	464
5	Medline	(bispectral index monitoring).ti,ab	865
6	Medline	(1 OR 2 OR 3 OR 4 OR 5)	97669
7	Medline	(burst suppression).ti,ab	2192
9	Medline	(prognostication).ti,ab	5338
10	Medline	(prognosis).ti,ab	297714
11	Medline	PROGNOSIS/	420680
12	Medline	(9 OR 10 OR 11)	599993
13	Medline	(6 AND 12)	866
14	Medline	(7 AND 12)	170
15	Medline	(cardiac arrest).ti,ab	29498
16	Medline	(cardiac arrest OR cardiopulmonary arrest OR circulatory arrest OR sudden cardiac arrest OR SCA OR sudden cardiac death OR SCD).ti,ab	44914
17	Medline	"HEART ARREST"/ OR "OUT-OF-HOSPITAL CARDIAC ARREST"/ OR "DEATH, SUDDEN, CARDIAC"/	40179

18	Medline	(out-of-hospital cardiac arrest OR OHCA).ti,ab	4366
19	Medline	(15 OR 18)	29557
20	Medline	(16 OR 17 OR 19)	82055
21	Medline	(13 AND 20)	17
22	Medline	(14 AND 20)	39
23	EMBASE	(bispectral index).ti,ab	3347
24	EMBASE	(BIS).ti,ab	75416
25	EMBASE	(bispectral index monitor).ti,ab	100
26	EMBASE	(bispectral index monitoring).ti,ab	374
27	EMBASE	(burst suppression).ti,ab	1576
28	EMBASE	(prognostication).ti,ab	8062
29	EMBASE	(prognosis).ti,ab	427109
30	EMBASE	(cardiac arrest).ti,ab	38295
31	EMBASE	(cardiac arrest OR cardiopulmonary arrest OR circulatory arrest OR sudden cardiac arrest OR SCA OR sudden cardiac death OR SCD).ti,ab	81792
32	EMBASE	(out-of-hospital cardiac arrest OR OHCA).ti,ab	6380
33	EMBASE	"BISPECTRAL INDEX"/	3596
34	EMBASE	(23 OR 24 OR 33 OR 25 OR 26)	77463
35	EMBASE	ELECTROENCEPHALOGRAM/	87053
36	EMBASE	(27 OR 35)	87745

37	EMBASE	PROGNOSIS/ OR "PROGNOSTIC ASSESSMENT"/	506930
38	EMBASE	(28 OR 29 OR 37)	692090
39	EMBASE	exp "HEART ARREST"/	69264
40	EMBASE	(30 OR 32)	38434
41	EMBASE	(31 OR 39 OR 40)	109823
42	EMBASE	(34 AND 38)	277
43	EMBASE	(36 AND 38)	3451
44	EMBASE	(41 AND 42)	36
45	EMBASE	(41 AND 43)	322
46	EMBASE	(27 AND 38)	217
47	EMBASE	(41 AND 46)	70
