# Supplementary material for: Role of bispectral index monitoring and burst suppression in prognostication following out-of-hospital cardiac arrest: a systematic review protocol
Source: Syst Rev. 2017 Sep 25;6:191. doi: 10.1186/s13643-017-0584-6 (PMC5613623; doi:10.1186/s13643-017-0584-6)
Supplement: Supplementary file 3 — Cochrane public health group data extraction and assessment template (DOCX 93 kb) [file 13643_2017_584_MOESM3_ESM.docx]

Appendix 3 Cochrane Public Health Group Data Extraction and Assessment Template

| **Study ID:** | **Report ID :** | Date form completed: |
| --- | --- | --- |
| First author: | Year of study: | Data extractor: |
| Citation: | | |

**1. General Information**

| Publication type Journal Article ⬜ Abstract ⬜ Other (specify e.g. book chapter)___________________ | |
| --- | --- |
| Country of study: | |
| Funding source of study: | Potential conflict of interest from funding? Y / N / unclear |

**2. Study Eligibility**

| **Study Characteristics** | | | | **Page/ Para/ Figure #** |
| --- | --- | --- | --- | --- |
| **Type of study**  (Review authors to add/remove designs based on criteria specified in protocol) | ⬜ Randomised Controlled Trial (RCT)  ⬜ Cluster Randomised Controlled Trial (cluster RCT) | | ⬜ Controlled Before and After (CBA) study   - Contemporaneous data collection - Comparable control site - At least 2 x intervention and 2 x control clusters |  |
|  | ⬜ Interrupted Time Series (ITS)   - At least 3 time points before and 3 after the intervention - Clearly defined intervention point | | ⬜ Other design (specify): |  |
|  | ⬜ A process evaluation of an included study design | | *Does the study design meet the criteria for inclusion?*  Yes ⬜ No ⬜ 🡪**Exclude** Unclear ⬜ |  |
|  | Description in text: | | |  |
| **Participants**  (Review authors insert inclusion criteria as defined in Protocol) | Describe the participants included: | | |  |
|  | Are participants defined as a group having specific social or cultural characteristics? | Yes ⬜ No ⬜ Unclear ⬜  Details: | |  |
|  | How is the geographic boundary defined? | Details:  Specific location (e.g. state / country): | |  |
|  | *Do the participants meet the criteria for inclusion?* | Yes ⬜ No ⬜ 🡪**Exclude** Unclear ⬜ | |  |

| **Types of intervention**  (Review authors insert inclusion criteria as defined in Protocol) | Strategies included in the intervention | |  | |  |
| --- | --- | --- | --- | --- | --- |
|  | Focus of the intervention | |  | |  |
|  | *Does the intervention meet the criteria for inclusion?* | | Yes ⬜ No ⬜ 🡪**Exclude** Unclear ⬜ | |  |
| **Duration of intervention** | Start date: | Stop date: | | Intervention duration: |  |
|  | *Is the duration of intervention adequate for inclusion?* | | Yes ⬜ No ⬜ 🡪**Exclude** Unclear ⬜ | |  |
| **Types of outcome measures**  (Review authors insert inclusion criteria as defined in Protocol) | List outcomes: | |  | |  |
|  | Outcome measured at a population level or individual level? | | Details: | |  |
|  | *Do the outcome measures meet the criteria for inclusion?* | | Yes ⬜ No ⬜ 🡪**Exclude** Unclear ⬜ | |  |
